# Mutations associated with autism lead to similar synaptic and behavioral alterations in both sexes of male and female mouse brain

**DOI:** 10.1038/s41598-023-50248-4

**Published:** 2024-01-04

**Authors:** Manish Kumar Tripathi, Shashank Kumar Ojha, Maryam Kartawy, Igor Khaliulin, Wajeha Hamoudi, Haitham Amal

**Affiliations:** Institute for Drug Research, School of Pharmacy, Faculty of Medicine, The Hebrew University of Jerusalem, Jerusalem, Israel

**Keywords:** Proteins, Molecular neuroscience

## Abstract

Autism spectrum disorder (ASD) is a neurodevelopmental disorder based on synaptic abnormalities. The estimated prevalence rate of male individuals diagnosed with ASD prevails over females is in a proportion of 4:1. Consequently, males remain the main focus in ASD studies in clinical and experimental settings. Meanwhile, some studies point to an underestimation of this disorder in females. In this work, we studied the sex differences of the synaptic and behavioral phenotypes of ASD mouse models. Juvenile male and female Shank3^Δ4–22^ and Cntnap2^−/−^ mutant mice and their WT littermates were used in the experiments. The animals were subjected to a Three-Chamber Sociability Test, then euthanized, and the whole cortex was used for the evaluation of the synaptic phenotype. Protein levels of glutamatergic (NR1) and GABAergic (GAD1 and VGAT) neuronal markers were measured. Protein level of synaptophysin (Syp) was also measured. Dendritic spine density in somatosensory neurons was analyzed by Golgi staining methods. Spine Density and GAD1, NR1, VGAT, and Syp levels were significantly reduced in Shank3^Δ4–22^ and Cntnap2^−/−^ mice compared to the control group irrespective of sex, indicating impaired synaptic development in the mutant mice. These results were consistent with the lack of differences in the three-chamber sociability test between male and female mice. In conclusion, female ASD mice of both mutations undergo similar synaptic aberrations as their male counterparts and need to be studied along with the male animals. Finally, this work urges the psychiatry scientific community to use both sexes in their investigations.

## Introduction

Autism spectrum disorder (ASD) is a prototypical form of a pervasive developmental disorder manifested by persistent abnormalities in social interactions, deficits in communication, and repetitive behavior^1,2^. Once developed, this disorder generates permanent lifelong disabilities^3^. The etiology of ASD is very complex and heterogeneous. It includes genetic, epigenetic, and environmental factors alone or in combination^4^. Males are 4 times more susceptible to being diagnosed with ASD as compared with females^5^. Furthermore, some studies revealed a higher threshold for reaching autistic affection status in females compared to males^6,7^. These observations strongly suggest sex dimorphism in the neurobiological, biochemical, morphological, and other manifestations of ASD.

The higher prevalence of males diagnosed with ASD can be at least partially explained by the effects of sex hormones. It has been suggested that males are inherently more vulnerable to the genetic or environmental risk factors of ASD whilst females are inherently protected from them^8^. It has been found that estrogen signaling can stimulate mitogen-activated protein kinase (MAPK) followed by the activation of the cAMP/PKA/CREB and PI3K/Akt protective pathways^9^, which may contribute to the suggested elevated threshold for ASD in females. It is also reported that the identified risk genes do not exhibit a discernible gender bias, with the exception of uncommon syndromic disorders^4^. Furthermore, no evidence suggests that males are at a higher risk than females due to their unique genetic source. Moreover, it has been observed that the role of de novo mutations in the etiology of ASD is more pronounced in females compared to males^10^.

Sex dimorphism in ASD has multiple manifestations, including changes in the immune and inflammatory systems, oxidative/nitrosative stress, metabolism etc.^8^. Thus, a proteomics study of human plasma has shown significant changes for a total of 12 proteins in ASD patients. Most of them appeared to be involved in the acute inflammatory response^11^. ASD has been linked to a number of abnormalities in the immune system, including maternal infection, cytokine, and chemokine activity, and aggravated cytokine and microglia responses^12^. It has been established that activation of the pro-inflammatory immune mechanism leads to dysregulation of glutamatergic neurotransmission activity in the brain, which is related to most ASD symptoms^13^. Overall, immune activation may be involved in male ASD bias, but the exact mechanisms remain obscure.

Studies on the cultured XX and XY rat neurons and 17-day-old rats showed much higher resistance to oxidative/nitrosative stress in females than in males^14^. Recently, we developed the SNOTRAP technology to study S-nitrosylation (SNO)-proteome^15,16^ and used this technique to show, for the first time, a significant increase in NO levels and a reprogramming of the SNO-proteome in a *Shank3* mouse model of ASD^15,17–21^. In our previous paper we found that both male and female mice showed increased of NO level. Inhibition of NO production led to reversal of the phenotype in both sexes^17^. In the other hand, we found significant differences in the NO and SNO-related biological functions between the cortices of male and female mice. The study revealed that in female mice, the SNO-enrichment of the synaptic processes was significantly increased, but in male mice, the SNO-dependent cytoskeletal processes were considerably elevated^22^. It can be suggested that the sex differences in the SNO-proteome may contribute to ASD pathogenesis.

**Figure 1:**
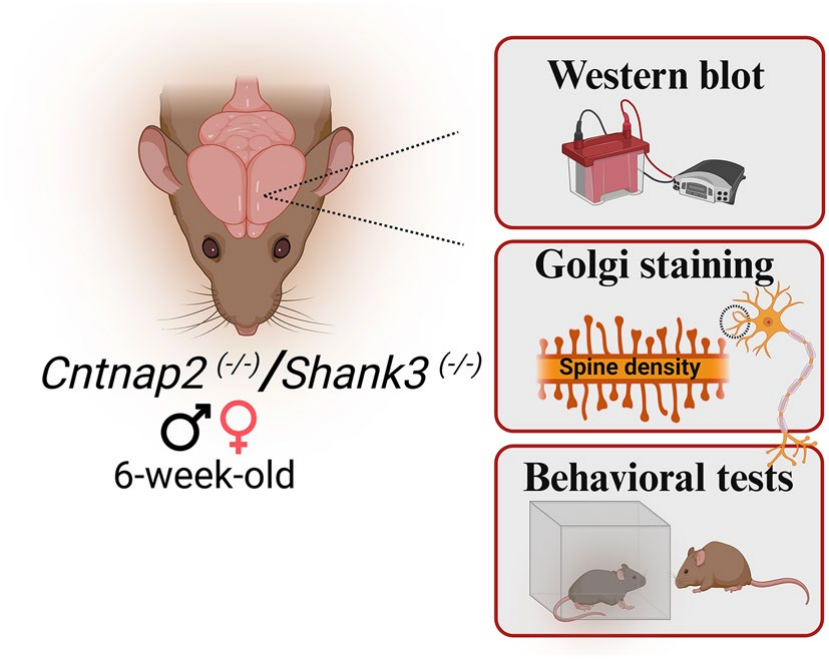
Schematic Workflow of Biochemical analysis, spine density, and behavioural tests performed in this study.

Biochemical, pharmacological, and behavioral studies indicate the possible involvement of glutamatergic and GABAergic systems in the ASD pathology^2^. Glutamatergic signaling is involved in the regulation of neuronal differentiation, migration, neurite outgrowth, neuron survival, and synaptogenesis in the brain development^2^. However, sex differences in the glutamatergic and GABAergic systems in ASD have not been well investigated^23^. In this work, we study the sex differences in the synaptogenesis, including the glutamatergic and GABAergic systems, in two transgenic mouse models of ASD, Shank3^−/−^ and Cntnap2^−/−^ (Fig. 1).

## Materials and methods

### Materials

Primary antibodies, anti-glutamate decarboxylase 1 (GAD1, #41318), anti-β-Actin (#3700), anti-synaptophysin (Syp, #36406), and secondary antibodies, HRP-conjugated anti-rabbit (7076S), HRP-conjugated anti-mouse (7074S), and protease phosphatase inhibitor cocktail (#5872) were purchased from Cell Signaling Technology (Danvers, MA, USA). Primary antibodies, NMDAR1 (ab109182), and SLC32A1/VGAT (1b235952) were procured from Abcam, Cambridge, UK. Other general chemicals were purchased from Sigma Aldrich (St. Louis, MO, USA) and Bio-Rad Laboratories (Haifa, Israel).

### Animal housing and tissue dissection

All animal experiments were conducted under the guidelines of the Institutional Animal Care committee of the Hebrew University of Jerusalem and use Committee and the Association for Assessment and Accreditation of Laboratory Animal Care International. The ethical approval number for this study is IACUC- MD-20-16049-3. This study is reported in accordance with ARRIVE guidelines.

6–8-week-old male and female Shank3^Δ4–22^ (4–22 exon deletion, denoted as Shank3^−/−^) (JAX stock #032169)^24^ and their wild-type (WT) littermates (used as control) were employed in the experiments. Also, Cntnap2^(−/−)^^25^ (JAX stock #017482) and their respective WT/control, C57BL/6 mice (Jax stock #000664), were used in this study. The animals were kept at the temperature of 23 °C in a 12-h light/dark cycle and fed ad libitum with standard mouse chow and water. For Western blot (WB) analysis, the entire cortex was isolated, snap-frozen in liquid nitrogen, and stored at − 80 °C, as described previously^26^.

### Three-chambered sociability test

A three-chamber sociability test was performed as described previously^17^. The social tests consisted of three sessions using a three-chambered box measuring 20 × 40 × 20 cm^59^. Two cylindrical wire cages were placed in the left and right chambers. In the first session (habituation), the test mouse was introduced to the center chamber and allowed to move freely over the testing area with all three chambers for 5 min. On the following day (the sociability session), one novel mouse (age and sex-matched) was introduced into one of the wire cages while the other cage remained empty. On the third day (the social memory session), a novel mouse (age and sex-matched) was introduced. The movements of the test mouse were recorded for 10 min during the sociability and the social novelty sessions. The interaction was calculated by tracking the nose point. All behavioral experiments were recorded and data are scored automatically through the video tracking software Ethovision XT 16 (Noldus Information technology BV).

### Western blots

The mice used in behavioural tests were sacrificed, and cortical tissues were isolated. The tissues were homogenized in a freshly prepared RIPA buffer containing 30 mM HEPES (pH 7.4), 150 mM NaCl, 1% Nonidet P-40, 0.5% sodium deoxycholate, 0.1% sodium dodecyl sulfate, 5 mM EDTA, 1 mM Na3VO4, 50 mM NaF, 1 mM PMSF, and 1% protease/phosphatase inhibitors cocktail (pH 7.7). All were purchased from Sigma Aldrich. The homogenization process was performed on ice using a Teflon pestle and a Jumbo Stirrer from Thermo Fisher Scientific, Waltham, MA, USA. The homogenates underwent centrifugation at 17,000 rpm for a duration of 30 min at a temperature of 4 °C. The supernatant of the sample was collected, and the concentration of protein was determined using the Bicinchoninic Acid (BCA) Protein Assay provided by Sigma Aldrich. The samples underwent polyacrylamide gel electrophoresis, after which they were transferred onto a PVDF membrane using a wet transfer method (Bio-Rad Laboratories). Non-specific sites were effectively inhibited by the addition of either 5% dried skimmed milk or 5% bovine serum albumin in Tris-buffered saline with Tween 20 (TBST). The TBST solution consisted of 135 mM NaCl, 50 mM Tris, and 0.1% Tween 20, with a pH of 7.4. This blocking process was carried out for a duration of 2 h at room temperature. PVDF membranes containing transferred proteins were incubated overnight at 4 °C on a shaker with a primary antibody. Primary antibodies used are anti-Syp (diluted at a ratio of 1:1000), anti-NMDA receptor 1 (NR1; diluted at a ratio of 1:1000), anti-GAD1 (diluted at a ratio of 1:1000), anti-vesicular GABA transporter (VGAT; diluted at a ratio of 1:1000), and anti-β-Actin (diluted at a ratio of 1:1000). Following the exposure to primary antibodies, the membranes underwent a washing step with TBST and subsequently underwent an incubation process with anti-mouse/rabbit HRP-conjugated secondary antibody for a duration of 1 h at ambient temperature . The specific binding of the protein under investigation was identified using an ECL substrate manufactured by Bio-Rad Laboratories. The bands were observed using the Bio-Rad Chemidoc imaging system, manufactured by Bio-Rad Laboratories (Hercules, California, United States), as described previously^17^.

### Golgi staining protocol

The Golgi staining technique and spine counting method were conducted in accordance with the procedures outlined in our previous study. A total of 9 mice per group, were used for this experiment. The Golgi staining procedure for the mouse brain was carried out according to the instructions provided in the user handbook for the FD Rapid GolgiStain™ Kit (FD NeuroTechnologies, Columbia, MD, USA). In summary, the mice underwent intracardial perfusion with a solution of 0.9% saline. The brain was dissected. A dissection was performed on the brain. The brains that were dissected had an initial immersion in the impregnation solution (solution A + B) for a duration of 21 days under the dark. Subsequently, they were incubated in solution C for a period of 3–7 days prior to being sliced. The Vibratome instrument was employed to produce coronal sections of mouse brains with a thickness of 100 µm. Following the preparation of the slides, the sections were subjected to incubation in staining solutions (namely, solution D + E) for a duration of 10 min. Subsequently, the stained sections were rinsed twice with distilled water. Subsequently, the samples underwent dehydration by a series of consecutive rinses in ethanol solutions with concentrations of 50, 75, 95, and 100%. Following this, the samples were subjected to clearing using xylenes and ultimately mounted using a mounting media. The images were captured using a Nikon-TL confocal microscope. In order to measure the spine density, a single dendritic branch from each of the four neurons was randomly chosen from the somatosensory cortex area. The density of dendritic spines was quantified utilizing the Image J program as described previously^17^.

### Statistical analysis

Statistical analysis was performed using Prism 9.3 (GraphPad Software, San Diego, CA). Data are presented as mean ± SEM. For the group comparisons, a one-way ANOVA test followed by the Bonferroni’s post hoc multiple comparison test was used. Statistical details used can be found in the figures and figure legends.

## Results

### Shank3^Δ4–22^ mutation leads to reduced synaptic protein expression in the cortex of both male and female mice

**Figure 2:**
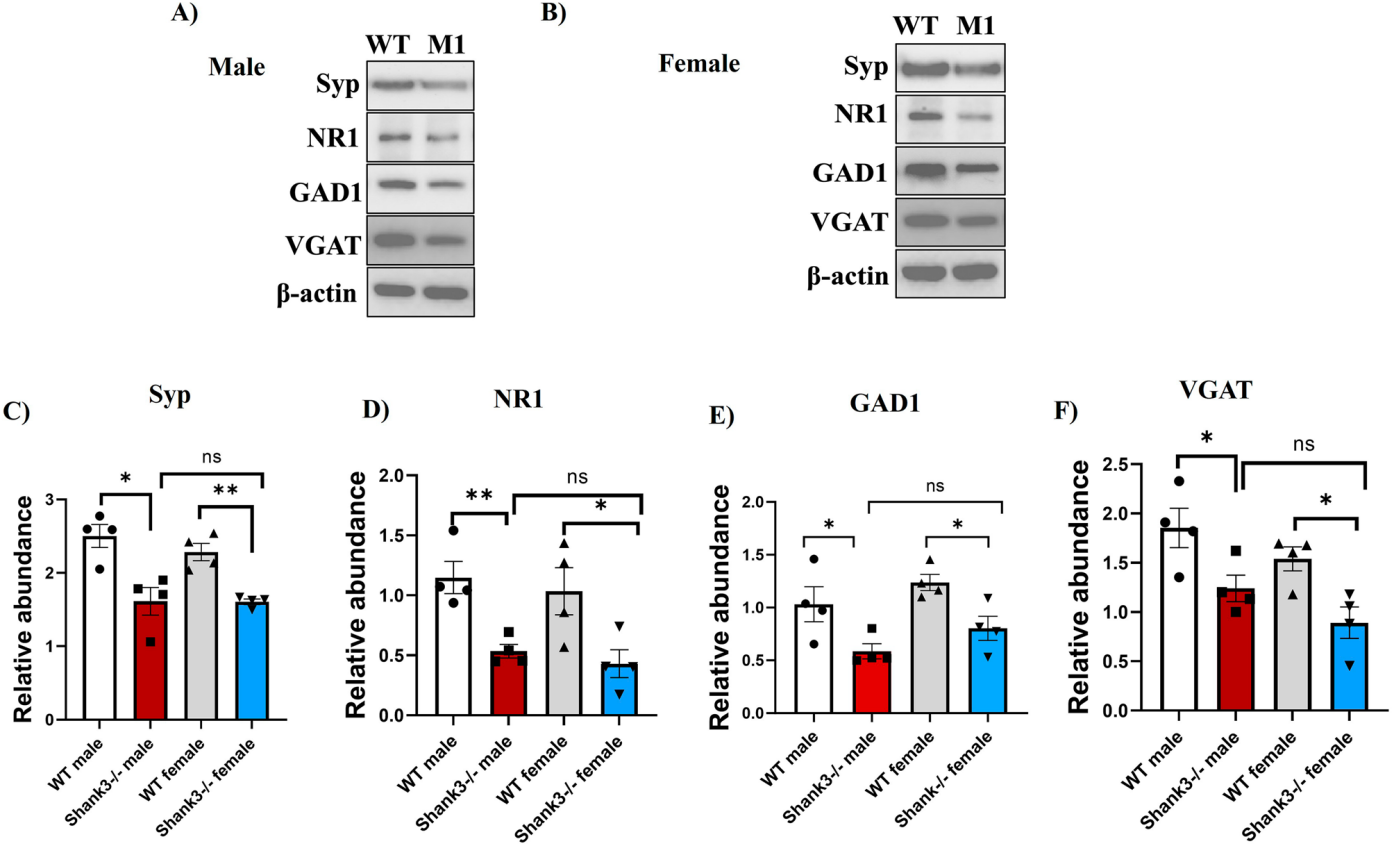
Synaptic, glutamatergic and GABAergic proteins reduction in both male and female Shank3^−/−^ mouse models of ASD. (**A** and **B**) Representative western blots of synaptic proteins Syp, NR1, GAD1, VGAT, in Shank3^−/−^ and WT mice. β-actin was used as a reference for protein loading. (**C**) Statistical analysis of the relative abundance of Syp proteins in all groups, [F_3,12_ = 0.6369]. (**D**) Statistical analysis of the relative abundance of NR1 proteins in all groups, [F_3,12_ = 1.602]. (**E**) Statistical analysis of the relative abundance of GAD1 proteins in all groups, [F_3,12_ = 0.6422). (**F**) Statistical analysis of the relative abundance of VGAT proteins in all groups, [F_3,12_ = 0.2450]. One-way Anova along with the Bonferroni multiple comparison tests was used for the western blot analyses in all groups. *p < 0.05, **p < 0.01, ***p < 0.001. n = 4 in all groups.

In Shank3^Δ4–22^ mice of both sexes, the synaptic marker protein, Syp levels were significantly reduced (Fig. 2A–C). The levels of glutamatergic marker NR1 (Fig. 2A, B and D), GABAergic markers GAD1 (Fig. 2A, B and E), and VGAT (Fig. 2A, B and F) were reduced as compared to WT littermates. However, no significant difference was seen in all proteins levels between male and female mice. Also, no statistically significant differences are observed in all protein expression between male and female WT mice either.

### Cntnap2^−/−^ mutation leads to reduced synaptic protein expression in the cortex of both male and female mice

**Figure 3:**
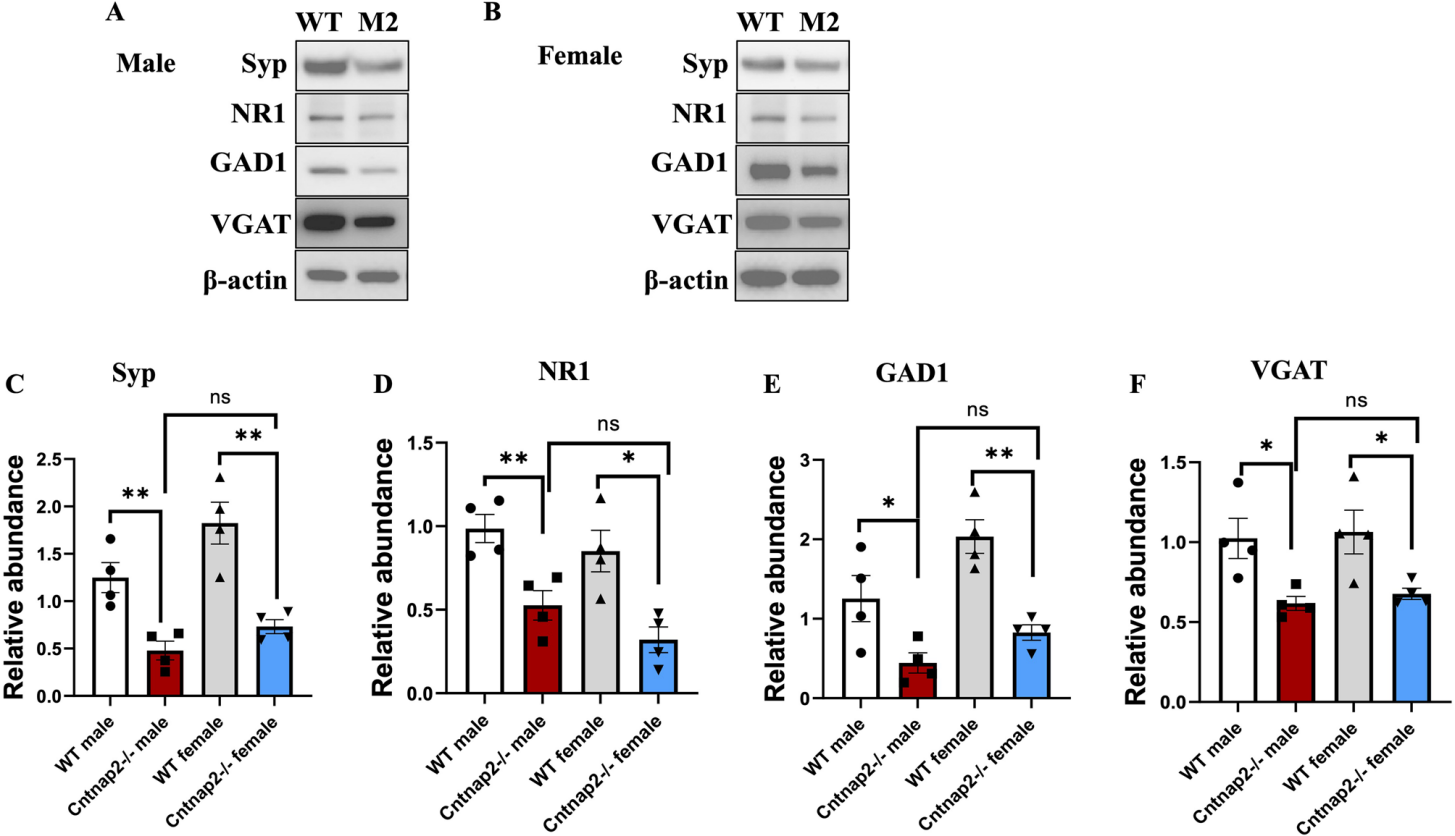
Synaptic, glutamatergic and GABAergic proteins downregulation in both male and female Cntnap2^−/−^ mouse models of ASD. (**A** and **B**) Representative western blots of synaptic proteins Syp, NR1, GAD1, VGAT, in Cntnap2^−/−^ and WT mice. β-actin was used as a reference for protein loading. (**C**) Statistical analysis of the relative abundance of Syp proteins in all groups, [F_3,12_ = 1.513] (**D**) Statistical analysis of the relative abundance of NR1 proteins in all groups, [F_3,12_ = 0.1386]. (**E**) Statistical analysis of the relative abundance of GAD1 proteins in all groups, [F_3,12_ = 2.196]. (**F**) Statistical analysis of the relative abundance of VGAT proteins in all groups, [F_3,12_ = 0.90285.682]. One-way Annova along with the Bonferroni multiple comparison tests was used for the western blot analyses in all groups. *p < 0.05, **p < 0.01, ***p < 0.001. n = 4 in all groups.

In Cntnap2^−/−^ mice of both sexes, the synaptic marker protein Syp levels were significantly reduced (Fig. 3A-C). The levels of glutamatergic marker NR1 (Fig. 3A, B and D), GABAergic markers GAD1 (Fig. 3A, B and E), and VGAT (Fig. 3A, B and F) were reduced as compared to WT littermates. However, no significant difference was seen between male and female mice from Cntnap2^−/−^ group. Also, there was no statistically significant difference in these parameters between male and female WT mice either.

### Shank3Δ^4–22^ and Cntnap2^−/−^ mutations in male and female mice lead to reduced dendritic spine density in the somatosensory cortex region

**Figure 4:**
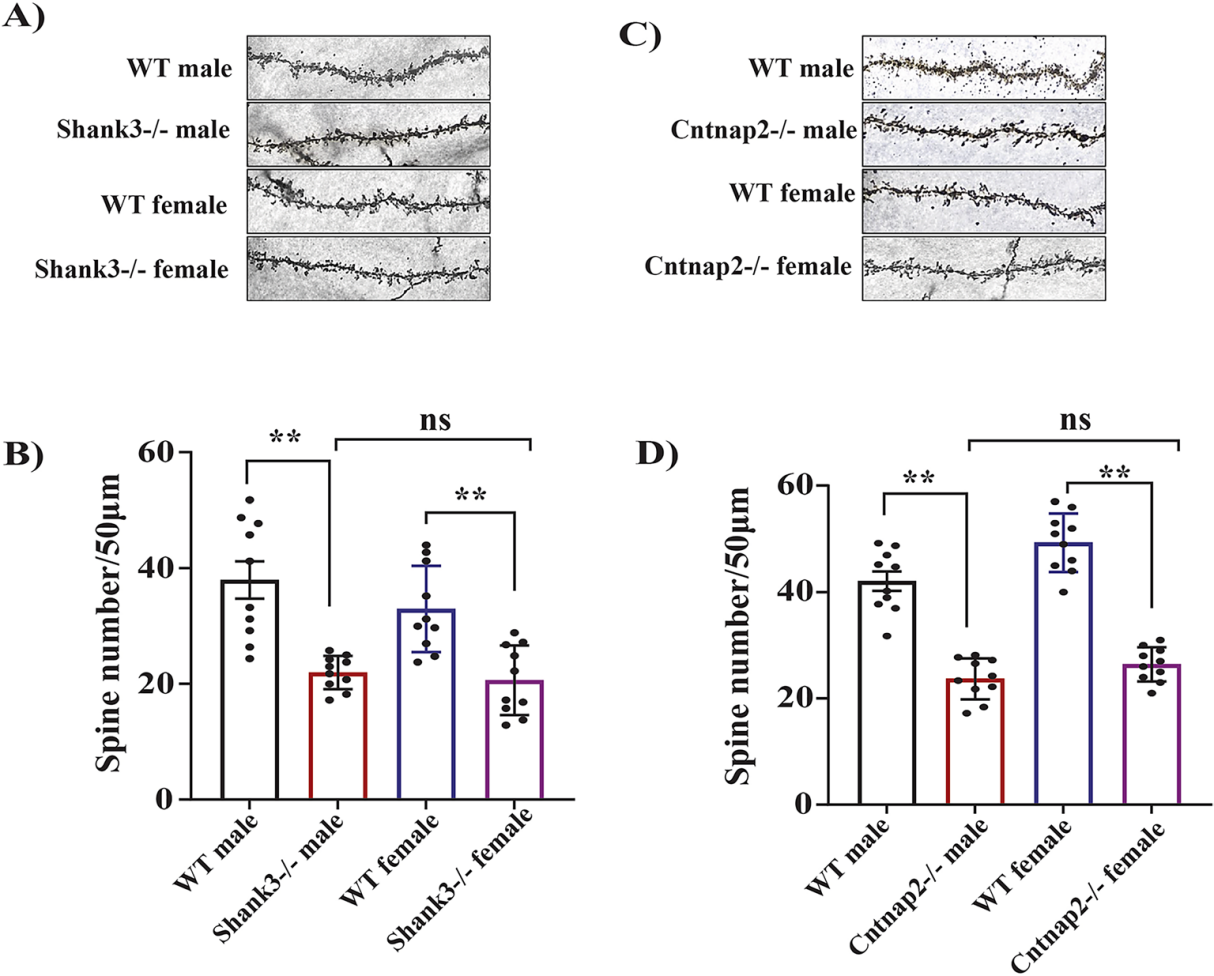
Dendritic Spine density reduction in both Shank3^−/−^ and Cntnap2^−/−^ group of mouse model of ASD in both sexes. (**A**) representative images of the dendritic spines from the male and female Shank3^−/−^ and WT brain; (**B**) statistical analysis of the dendritic spine density; [F_3,36_ = 6.921] (**C**) representative images of the dendritic spines from the male and female Cntnap2^−/−^ and WT brain; (**D**) statistical analysis of the dendritic spine density [F_3,36_ = 2.031]. One-way Anova along with the Bonferroni multiple comparison tests was used. * *p* < 0.05, ** *p* < 0.01, *** *p* < 0.001. n = 10.

Dendritic spines are the main sites for excitatory neuronal transmission. They were found to be reduced in both the Shank3^Δ4–22^ (Fig. 4A and B) and Cntnap2^−/−^ (Fig. 4 C and D) mouse models of ASD as compared to WT groups. A similar reduction was found in both sexes. We did not see any change in WT groups of both sexes.

### Shank3^Δ4–22^ and Cntnap2^−/−^ mutations in male and female mice affect the social interaction in the three-chamber sociability test

**Figure 5:**
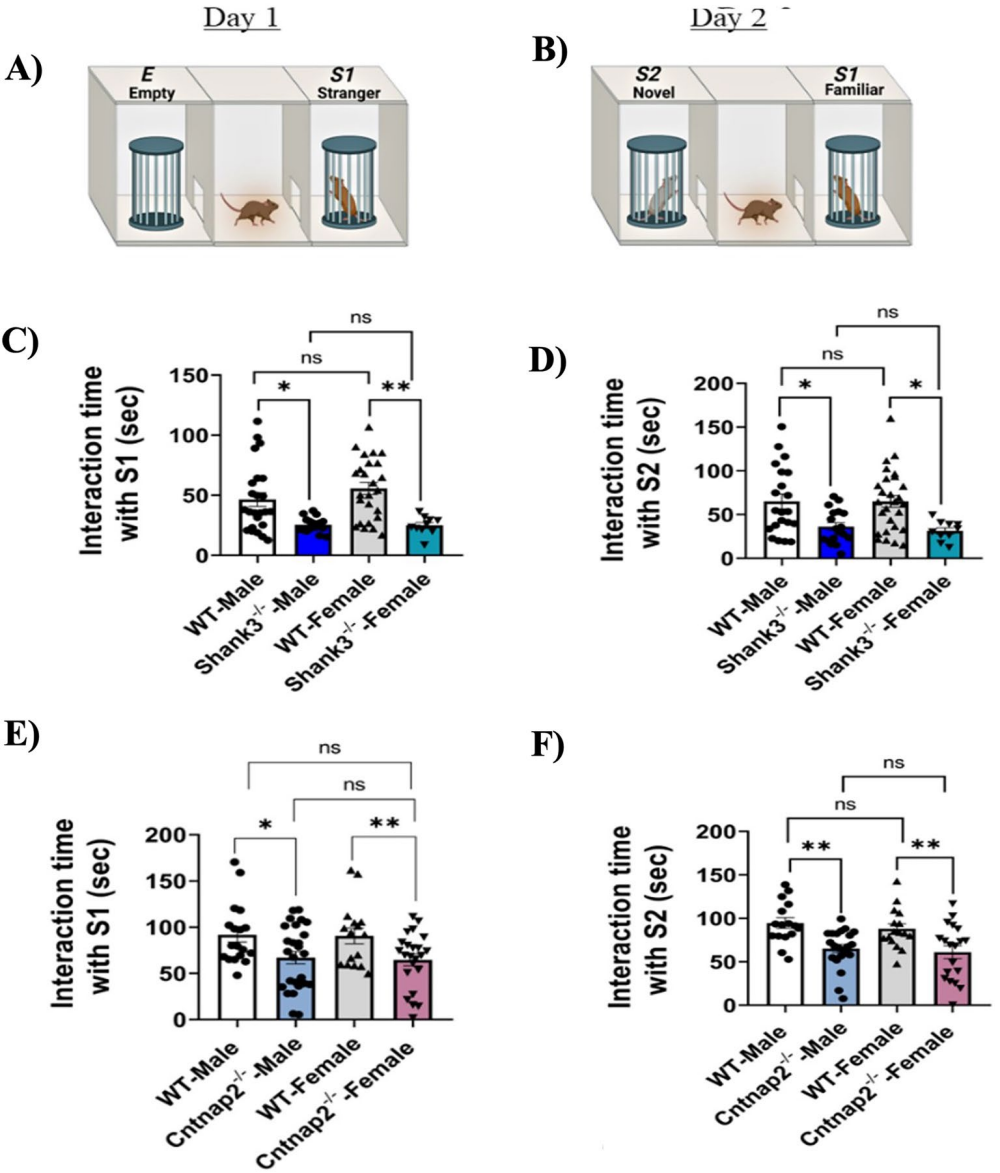
Social interaction deficits in the males and females of *Shank3*^*−/−*^ and *Cntnap2*^*−/−*^ mutant mice. (**A**) Three-chamber sociability test platform of day 1. (**B**) Three-chamber sociability test platform of day 2. (**C**) Statistical analysis of time interacting with a stranger mouse (S1). The *Shank3*^*−/−*^ male (N = 17) and female (N = 10) mice spent significantly less time interacting with the stranger mouse than their WT male (N = 24) and female (N = 25) littermates. *The data is presented as mean* ± *SEM*. One-way ANOVA followed by Bonferroni- comparison tests were used, *F*_*(*3,72)_ = 8.834. *P < 0.05, **P < *0.01, ns* = *not significant*. (**D**) Statistical analysis showing time interacting with a novel stranger mouse (S2). The Shank3^−/−^ male (N = 18) and female (N = 10) mice spent significantly less time interacting with the novel mouse than their WT male (N = 21) and female (N = 27) littermates. *The data is presented as mean* ± *SEM. One-way ANOVA followed by Bonferroni- comparison tests were used, F*_*(3,72)*_ = 8.834. *P < *0.05, ns* = *not significant*. (**E**) Statistical analysis of time interacting with S1. The *Cntnap2*^*−/−*^ male (N = 26) and female (N = 23) mice spent significantly less time interacting with the stranger mouse than their WT male (N = 18) and female (N = 16) counterparts*. The data is presented as mean* ± *SEM. One-way ANOVA followed by Bonferroni- comparison tests were used, F*_*(3,79)*_ = 5.033. *P < 0.05, **P < *0.01, ns* = *not significant*. (**F**) Statistical analysis of time interacting with S2. The *Cntnap2*^*−/−*^ male (N = 23) and female (N = 19) mice spent significantly less time interacting with S2 than the WT male (N = 16) and female (N = 16) mice. *The data is presented as mean* ± *SEM. One-way ANOVA followed by Bonferroni- comparison tests were used, F*_*(3,70)*_ = 8.685. **P < *0.01, ns* = *not significant*.

We conducted three-chamber sociability test to evaluate the mice tendency toward interacting with a stranger mouse. Mice typically prefer spending time with another mouse (sociability) and exhibit interest in investigating a new intruder more than a familiar one (social memory). This test involves two sessions: in the first one the test mice encounter a stranger mouse (S1) and an empty cage (E), assessing sociability (Fig. 5A- Left), and another session where mice encounter the first intruder (S1) and a new intruder (S2) to evaluate social memory (Fig. 5B- Right).

On the first session of the test, the Shank3^Δ4–22^ male and female mice spent significantly less time interacting with the stranger mouse (S1) compared to their WT male and female littermates (Fig. 5A and C), reflecting a lack of sociability among the Shank3^Δ4–22^ mice of both sexes. Similarly, the Cntnap2^−/−^ male and female mice showed a significant decrease in the interaction time with S1 compared to the male and female WT mice, exhibiting a reduced sociability among the Cntnap2^−/−^ mice of both sexes (Fig. 5E). On the second session of the test (Fig. 5B), the Shank3^Δ4–22^ male and females mice spent less time interacting with the intruder mouse (S2) compared to the WT male and female mice showing reduced social memory (Fig. 5D). Similar observations were found among the Cntnap2^−/−^ male and female mice, exhibiting also a reduced social memory (Fig. 5F). Results from these experiments show that there are no significant sex differences in social interactions among the mutant mice.

## Discussion

This study is focused on the sex differences of the impairments of synaptic development in two well-established models of ASD, Shank3^−/−^ and Cntnap2^−/−^ mice. Some reports showed the increased vulnerability of males to genetic and environmental risk factors of ASD^5^, and the elevated resistance to these factors in females^6,7^, which suggest the sex-dependent differences in the etiology and pathogenesis of ASD^27,28^. Indeed, differences between male and female autistic individuals have been found in the brain morphology^29,30^, and distribution of the white and grey matter^31^. Sex differences in ASD were also observed in the functional organization^32^, behavioral characteristics^33^, immune and inflammation systems^8^, NO production^22^, and oxidative/nitrosative stress^34^ etc. Some works point to the sex differences in synaptic development in ASD^23^. Intriguingly, our study revealed a significant reduction in the levels of proteins in both glutamatergic and GABAergic systems, delayed synaptic development manifested in the reduction of the spine density, and social behavior deficits in both Shank3^−/−^ and Cntnap2^−/−^ models of ASD. However, no significant differences were observed in these parameters between male and female autistic mice.

Synaptic developmental abnormalities are considered essential contributors to ASD pathology to such an extent that ASD has been referred to as “developmental synaptopathy”^35^. The glutamatergic and GABAergic synaptic transmission in the brain plays a special role in ASD pathogenesis^2^. It has been suggested that several behavioral deficits of ASD can be associated with the lack of long-term potentiation due to NMDA-type glutamate receptor dysfunction^2,36^. GAD isoform Gad1 or GAD 67 mRNA is found to be decreased in Purkinje cells of ASD patients compared to control^37^. However, the exact mechanisms that underlie the behavioral abnormalities in ASD related to dysregulation of glutamatergic and GABAeric systems remain obscure^2^. It has been established that glutamate-mediated neurotransmission is finely regulated by gonadal hormones^38^, which determine sex differences in normally developing brains^23^. Thus, morphological clinical studies have found that the levels of glutamate in the frontal grey matter, sensorimotor and anterior cingulate cortex, striatum, cerebellum, and basal ganglia increased in females compared to males^39–41^. Meanwhile, glutamate concentration in the parietal grey matter^41^ and prefrontal cortex (PFC)^42^ was found to be higher in men than in women. The sex differences were also found in glutamate concentration in blood plasma. In particular, higher glutamate levels were observed in males than females, and there was a negative correlation between the levels of glutamate and female steroids^43^. Similarly, significant sex differences in the glutamate system were found in small rodents^44^. Regarding the glutamate receptor distribution, female rats display increased levels of GluN1, Glu2B, mGlu5, and mGlu2/3 and the NMDAR subunits in the hippocampus^45,46^. The results of our experiments showed significantly reduced levels of the components of glutamatergic and GABAergic systems in the cortices of autistic mice.

Sex hormones are also involved in the regulation of the development of dendritic spines in many areas of the brain, and these regulatory mechanisms appear to be abnormal in several mouse models of ASD^47–49^ and ASD patients^50,51^. Overall, the above data indicate that ASD could differentially affect the glutamate and GABA systems in males and females. The results of our experiments showed a significant depletion of the components of glutamatergic and GABAergic systems accompanied by a reduction in dendritic spine number and density both in *Shank3*^*−/−*^ and *Cntnap2*^*−/−*^ mutant mice. The impaired synaptic development could be linked to the sociability deficits (the three-chamber sociability test) observed in these mice. Indeed, the association between the synaptic abnormalities and social behavior in ASD mouse models, including *Shank3* and *Cntnap2* mutant mice, and patients has been confirmed previously^52–54^. However, we did not find any sex differences in the synaptic phenotype and the levels of proteins of the glutamatergic and GABAergic systems in the cortex of the *Shank3*^*−/−*^ and *Cntnap2*^*−/−*^ mouse models. These results, however, do not rule out the sex differences in the glutamatergic and GABAergic systems in these ASD mice. It can be speculated that the differences could be found in a detailed investigation of the different brain regions of these animals. The evidence of significant sex differences is also observed in the nitric oxide (NO) production and oxidative/nitrosative stress in different brain regions. These differences were determined by both sex hormones and genes. NO is a signaling free radical molecule that regulates numerous physiological functions^55,56^, including glutamate metabolism in the brain^57^. Some scientists consider NO as an effector molecule in the determination of sex differences in development^58^. A study on neuronal NO synthase (nNOS)-deficient mice revealed that estradiol stimulates NO production via estrogen receptor β (ERβ)-mediated nNOS expression, whilst low estrogen attenuates local NO in the hippocampus^59^. Sex differences in nNOS mRNA were observed in the adult preoptic area (POA)/anterior hypothalamic region of rats^60^. The authors found a significant decrease in the nNOS mRNA expression in the rostral POA in female but not male rats. Scordalakes et al. have shown that the effects of the gonadal hormones on nNOS are different in different brain areas of mice. They also reported the sex differences in POA. In particular, they found that testosterone is acting via estrogen receptor-α to up-regulate and through androgen receptor to down-regulate nNOS-immunoreactivity cell numbers in this brain region^61^. Studies on the cultured XX and XY rat neurons and 17-day-old rats showed much higher resistance to oxidative/nitrosative stress in females than in males^14^. Interestingly, it has been shown that this phenotypic difference in the brain appears to be independent of gonadal phenotype^14,62^. Nevertheless, this does not exclude the effects of the gonadal hormones on the sex differences in resistance to oxidative/nitrosative stress. Thus, Raficov et al. have recently reported that the male gender in mice is associated with higher production of oxidants and lower activity of the antioxidant system due to the effects of testosterone^34^ and these results were in accord with others showing the role of this hormone in oxidative stress^63,64^.

Nevertheless, the fact that female autistic mice of the two models display the abnormal synaptic phenotype comparable to that of males is an important finding of this study. It opposes to some extent the view that females are protected from the impact of becoming autistic^65^ and suggests that ASD is characterized by significant impairments in glutamate/GABA neurotransmission both in male and female mice.

The generally accepted opinion that ASD is mostly diagnosed in males than in females^66^, which supports the “Extreme Male Brain” and “Female Protective Effect” theory. This suggests that many autistic traits are maleness pushed to the point of dysfunction, such as low empathy, excessive systematizing, poor bonding, and limited social skills^67^ and Female Protective Effect theory states that females have a lower risk of developing ASD compared to males^65^. However, relatively recent large-scale population studies have found that the relative prevalence of female subjects with this disorder is higher and the male-to-female ratio is 4:1^68,69^, or even lower^70^. The estimation of the number of males *vs.* females affected with ASD could depend on the factors taken into account for this calculation. For example, de Giambattista et al. have reasoned that along with biological factors, methodological factors could affect the recognition of ASD in females^33^. The majority of epidemiological studies were performed on males. These studies did not take into account the phenomena like “camouflaging”, which is observed mainly in female ASD patients^71^, and there were, in fact, very few female-oriented studies. In light of these data, the lack of sex difference in the synaptic phenotype of the autistic mice in our experiments confirms the risks of developing ASD both for males and females and that delayed and abnormal synaptogenesis could occur in individuals of both sexes. Many scientists argue that including female data in research protocols could hinder scientific studies since larger sample numbers would need to be obtained, and costs would increase. However, females are not more vary than males^72,73^. In neurological disorder research, there are more than five studies on males for every single study on females; about 20% of studies included both sexes, and 25% did not state which study subjects were male or female^74,75^. The spontaneous behavior of female mice is only minimally affected by the estrous state, with both females and males demonstrating highly individualized exploration patterns and female spontaneous behavior being less variable than male behavior^76^. Female subjects are often underrepresented in animal research, but recent studies indicate that unstaged female rodents show no higher variability than males. Utilizing factorial designs enables the analysis of both sexes without requiring larger sample sizes, and presenting sex differences should include information on size effect^72^.

In conclusion, female ASD mice undergo similar synaptic and behavioral aberrations as their male counterparts and need to be studied along with the male animals. Finally, this work urges the psychiatry scientific community to use both sexes in their investigations (“Supplementary information”).

### Supplementary Information

Supplementary Information.

## Data Availability

Upon request from the corresponding author.
