# Supplementary material for: Mutations associated with autism lead to similar synaptic and behavioral alterations in both sexes of male and female mouse brain
Source: Sci Rep. 2024 Jan 4;14:10. doi: 10.1038/s41598-023-50248-4 (PMC10766975; doi:10.1038/s41598-023-50248-4)

All Original western blot uncropped images

Shank3 male group

Shank3 Male SYP

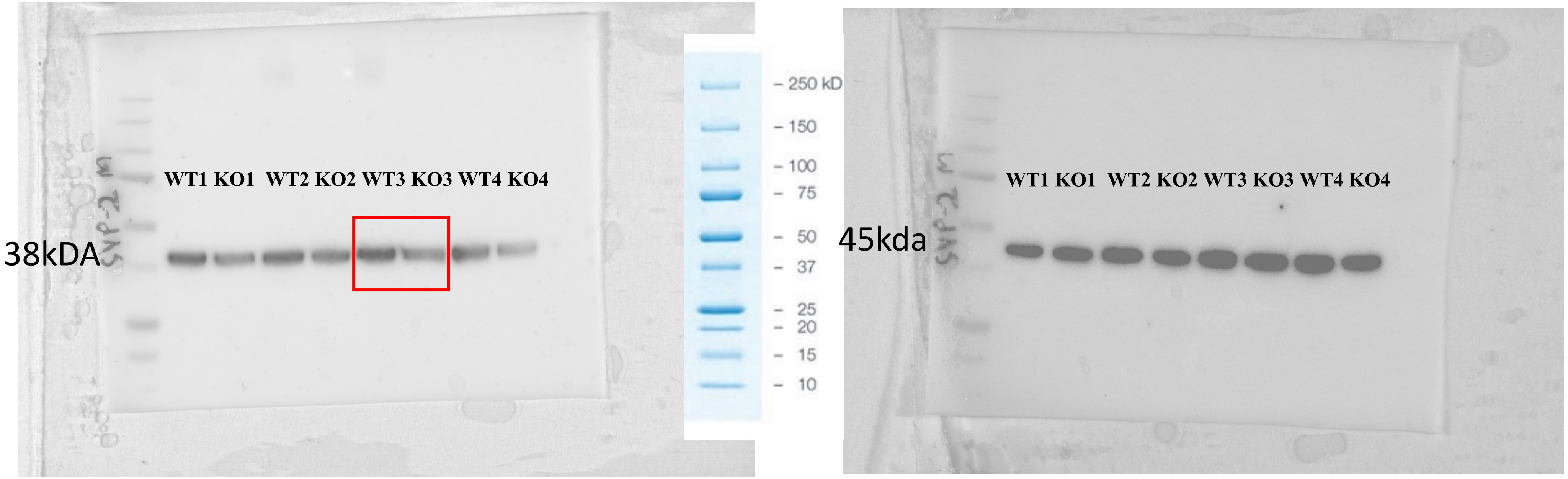

Shank3 Male VGAT

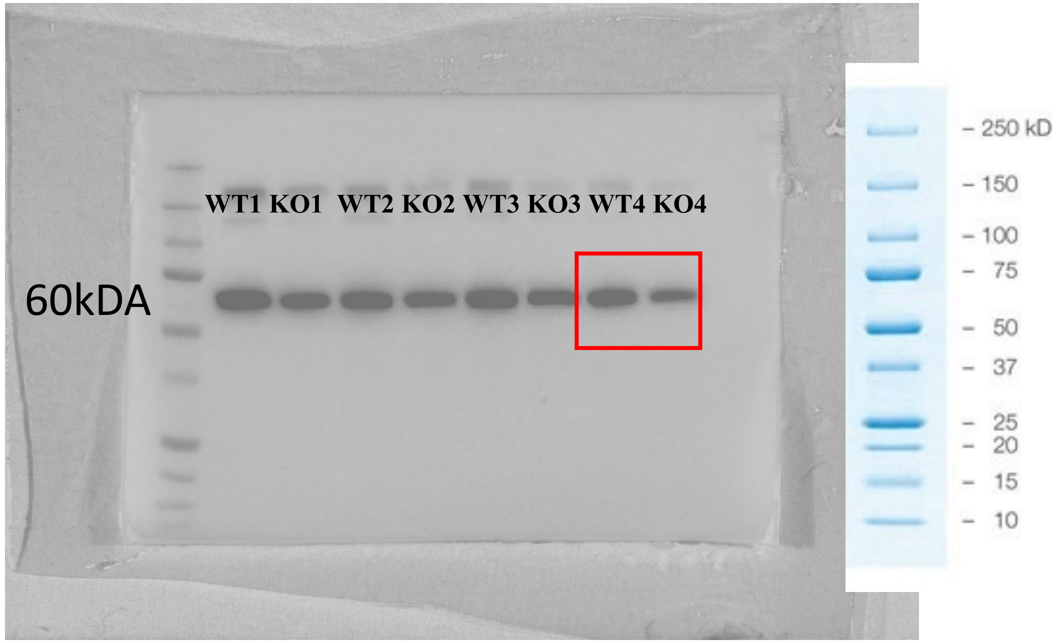

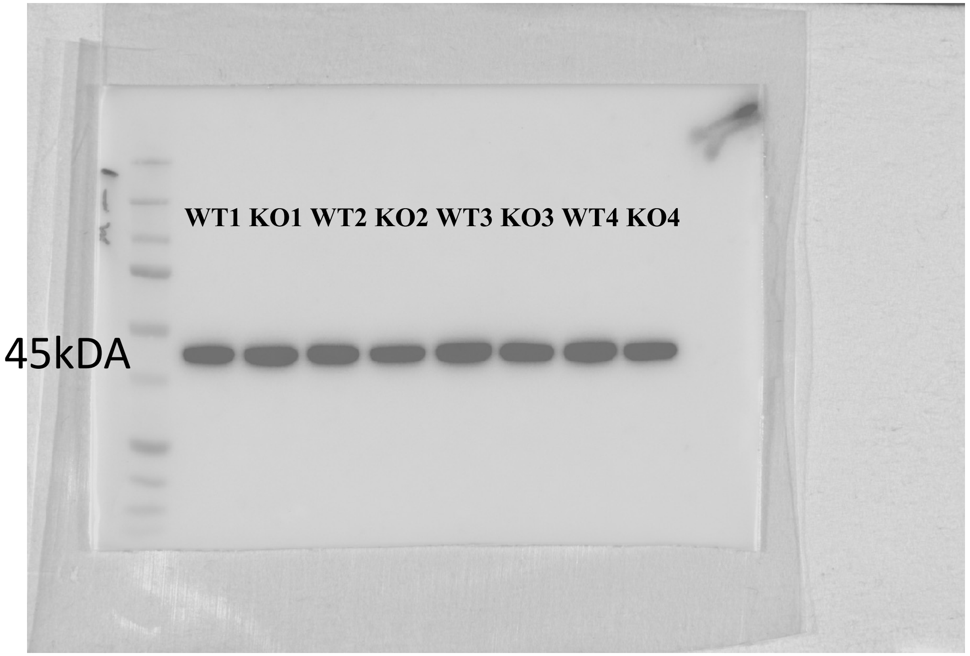

NR1 Shank3 male

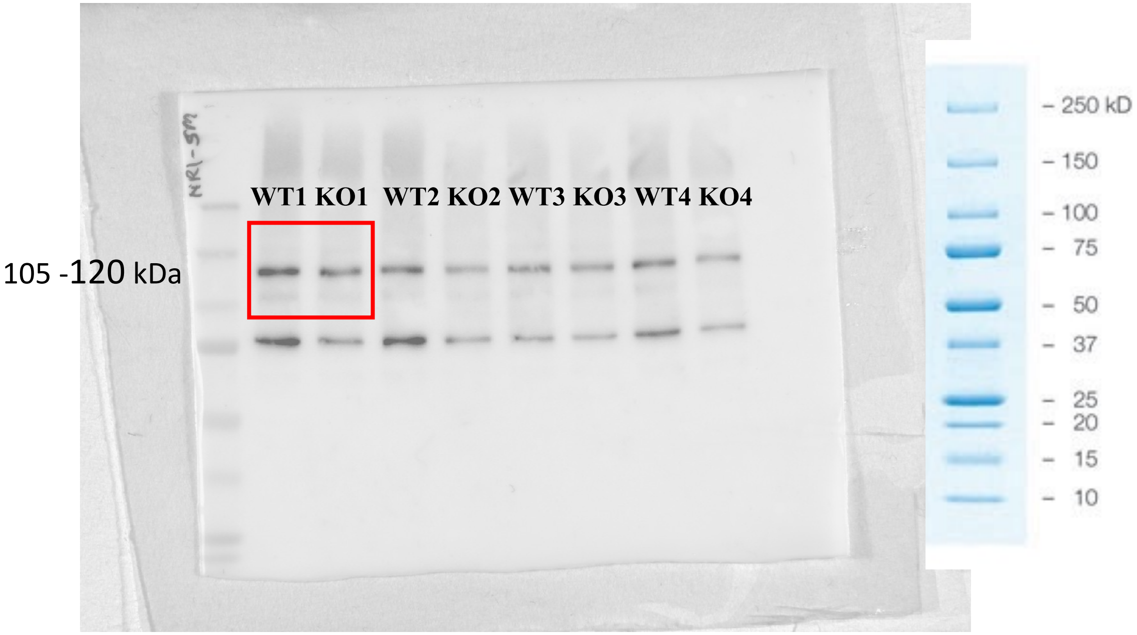

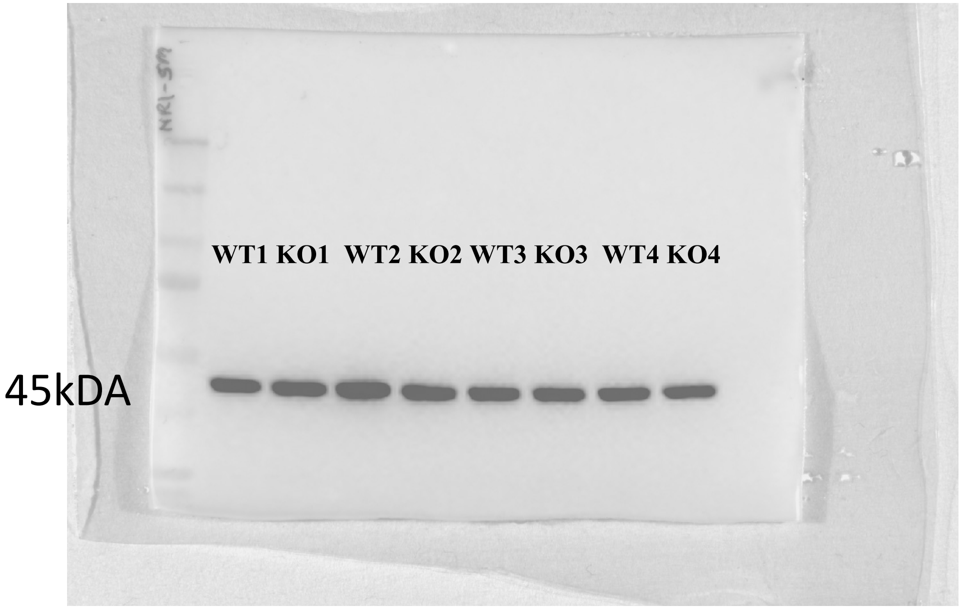

GAD1 Shank3 male

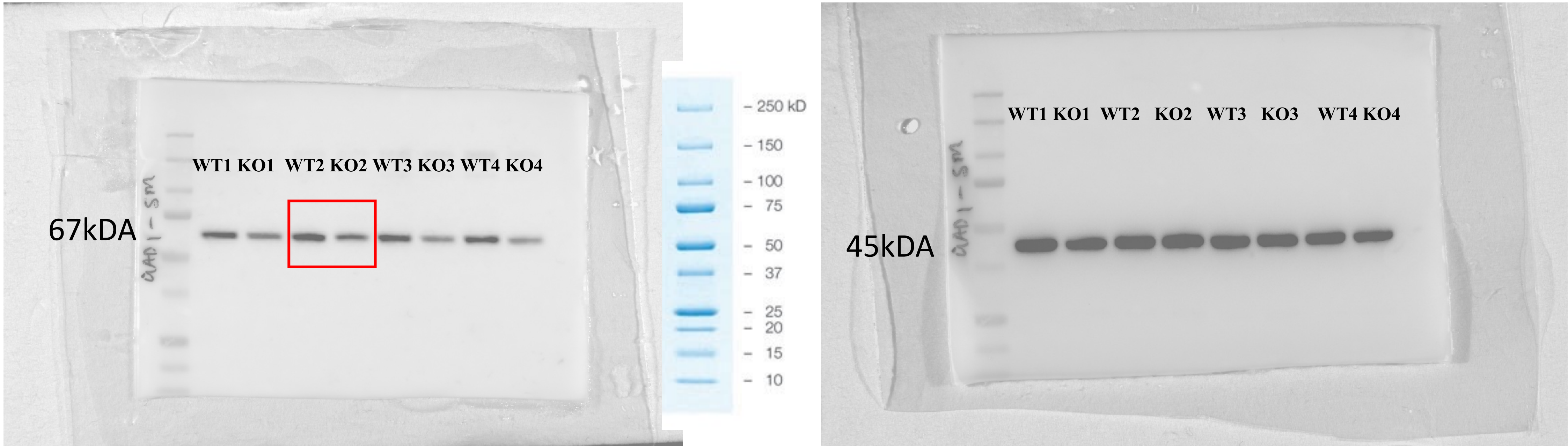

Cntnap2 Male group

Cntnap2 Male SYP

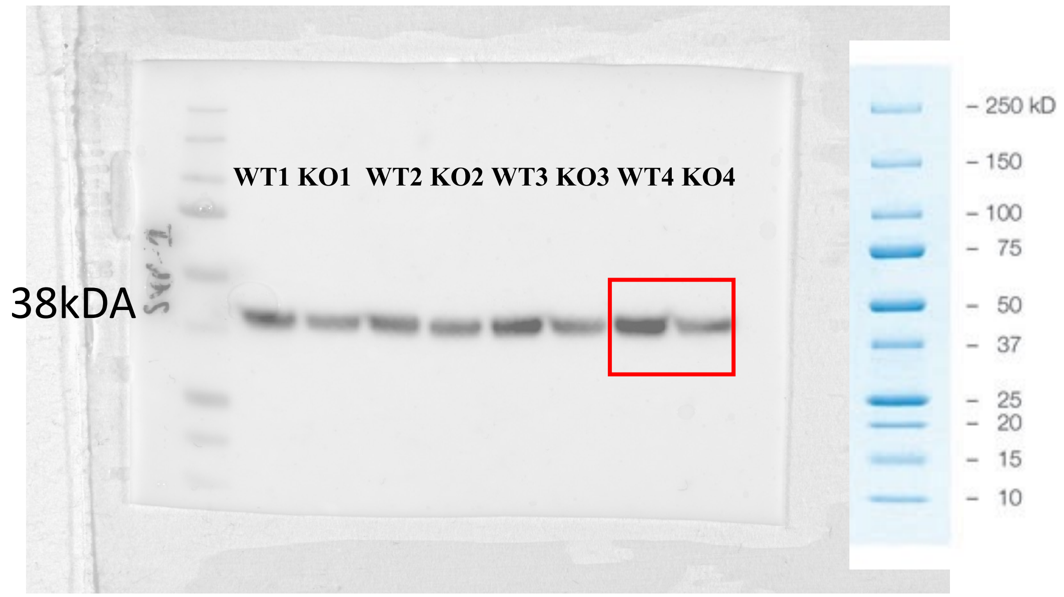

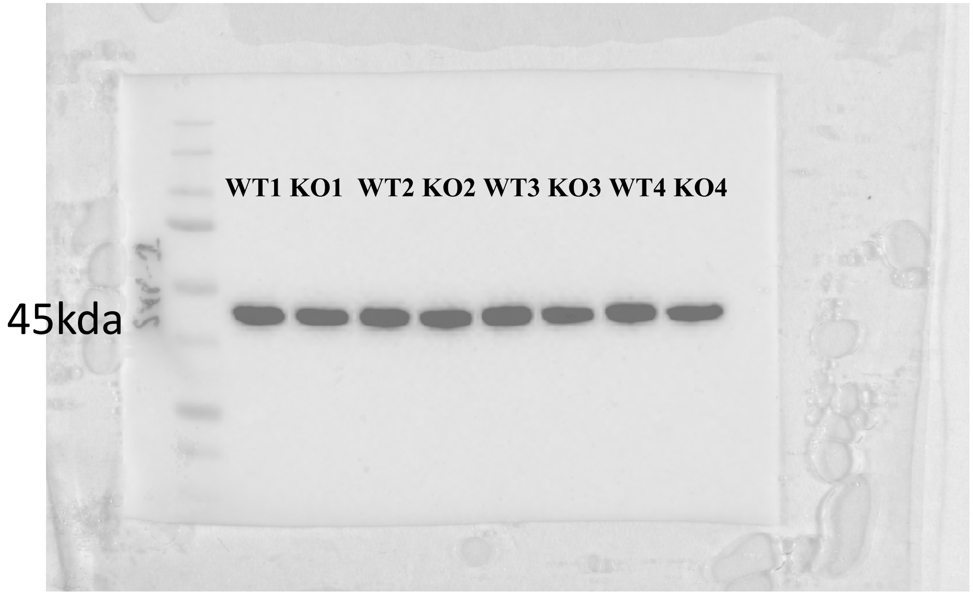

GAD1 Cntnap2 Male

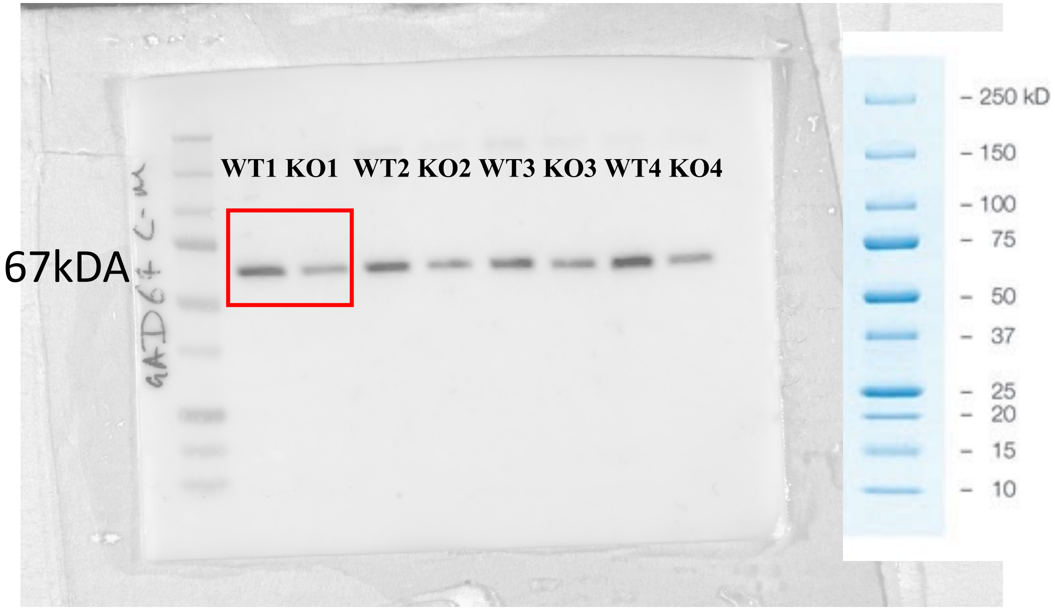

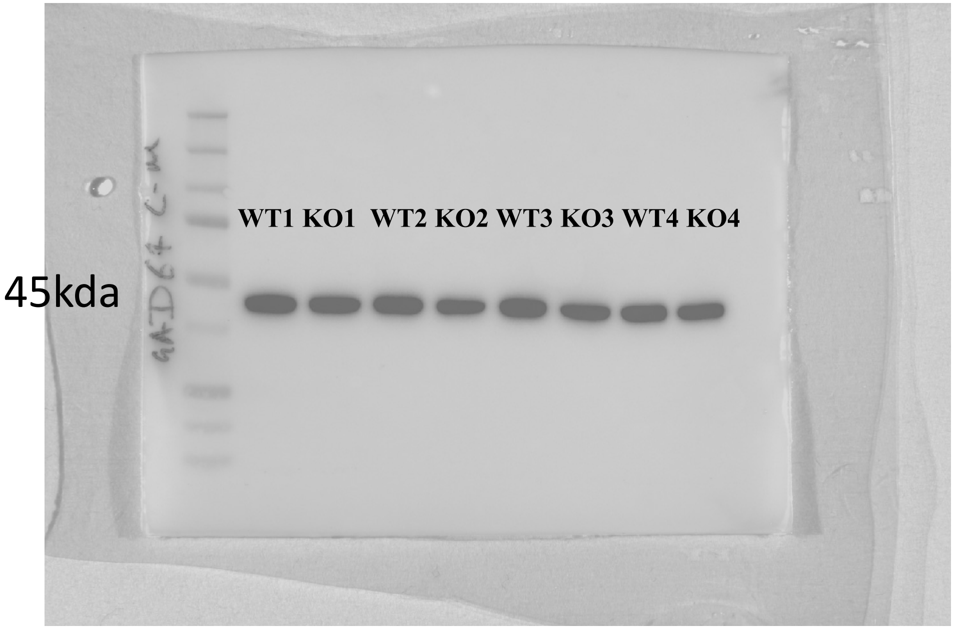

NR1 Cntnap2 Male

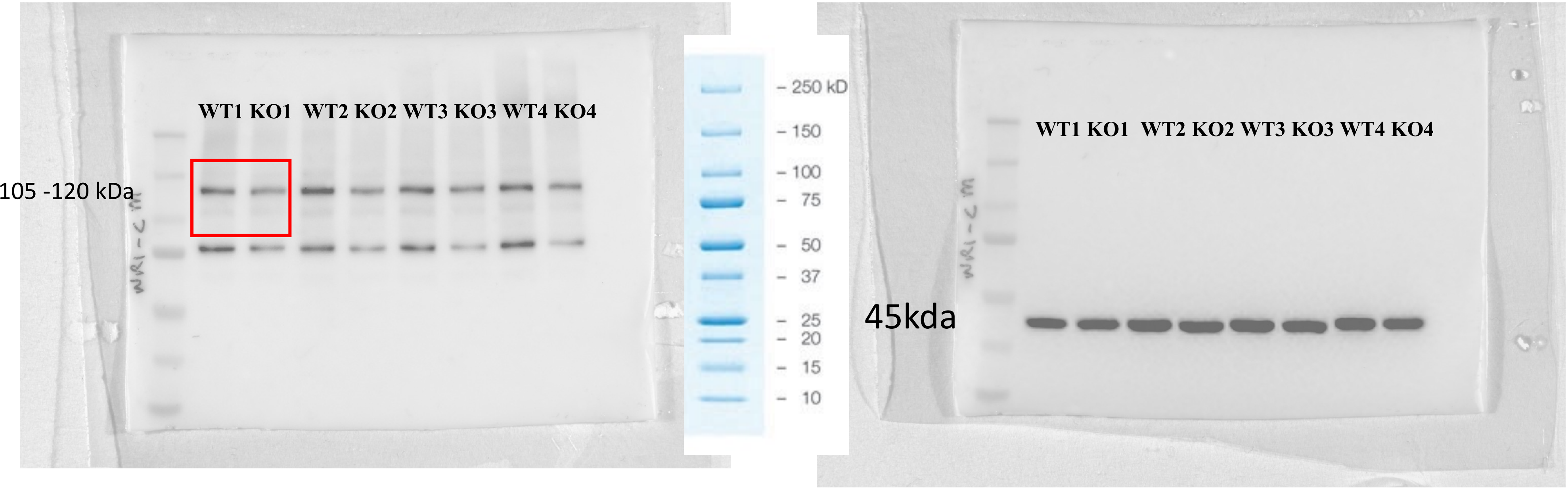

VGAT Cntnap2 Male

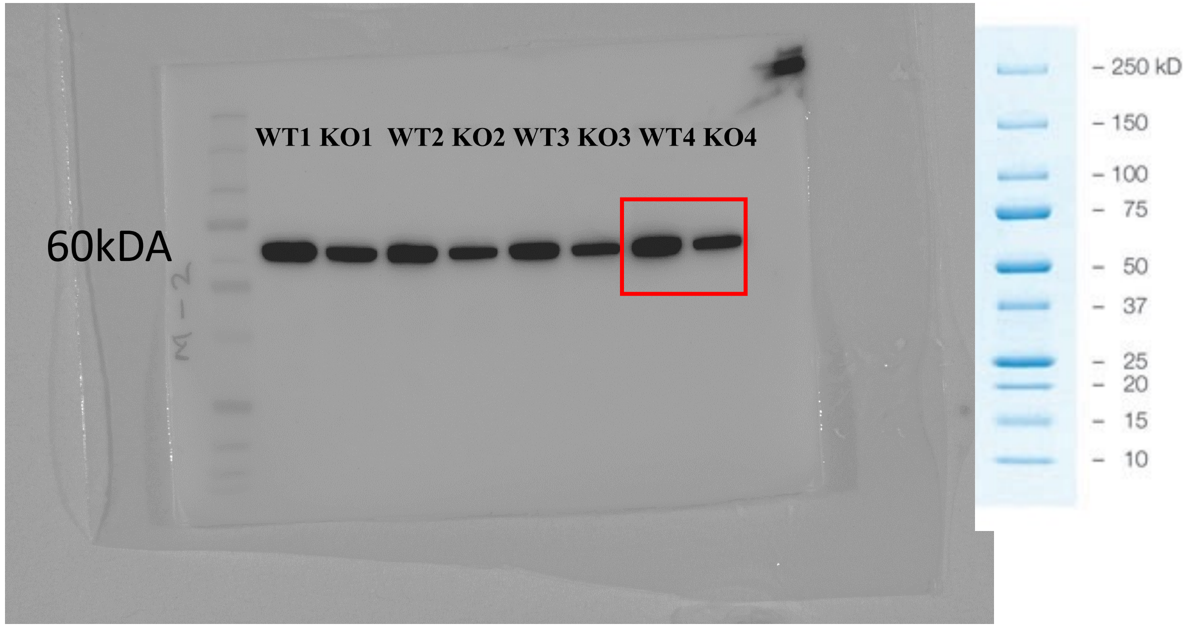

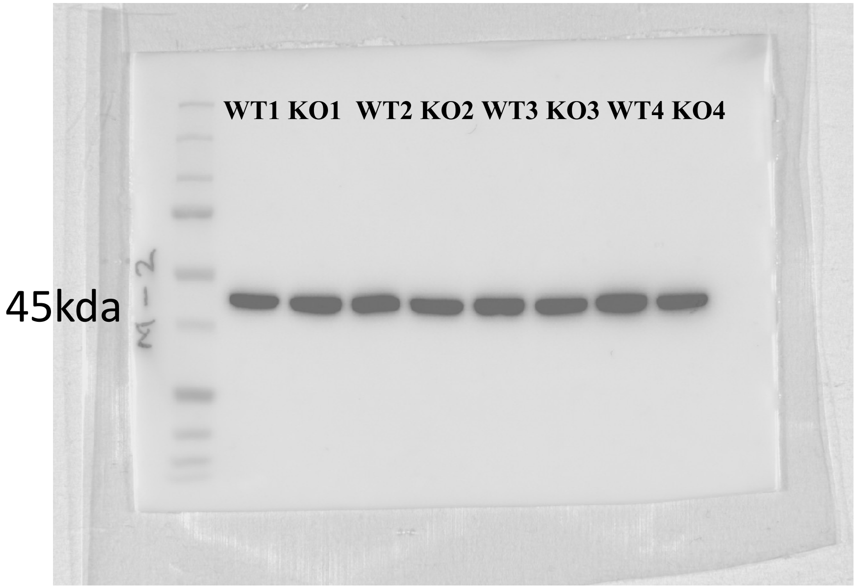

Shank3 Female group

Shank3 Female SYP

38kDA

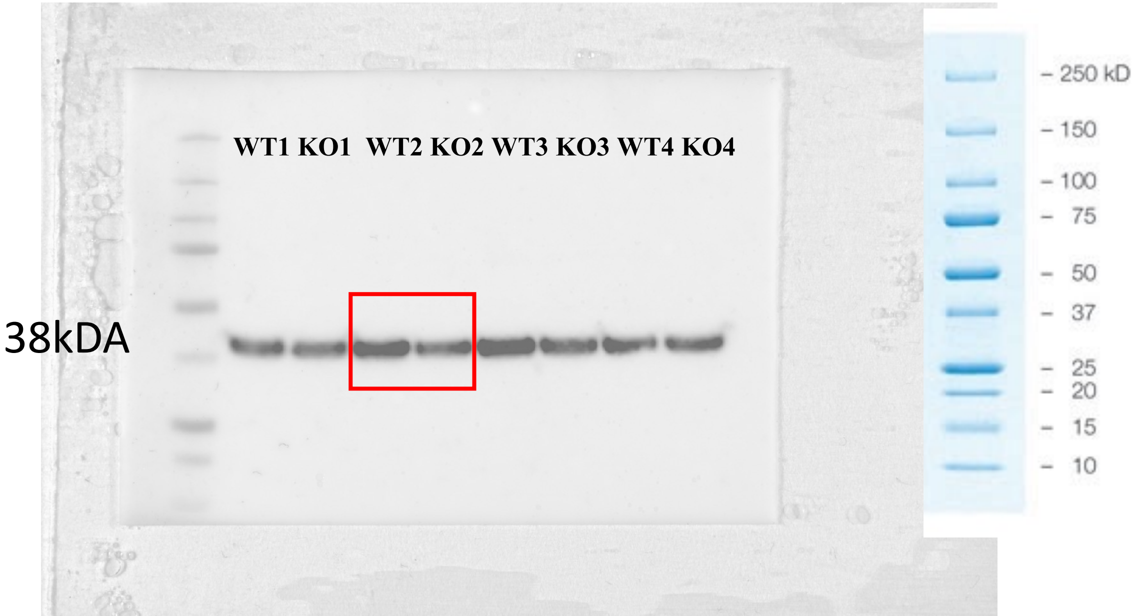

45kda

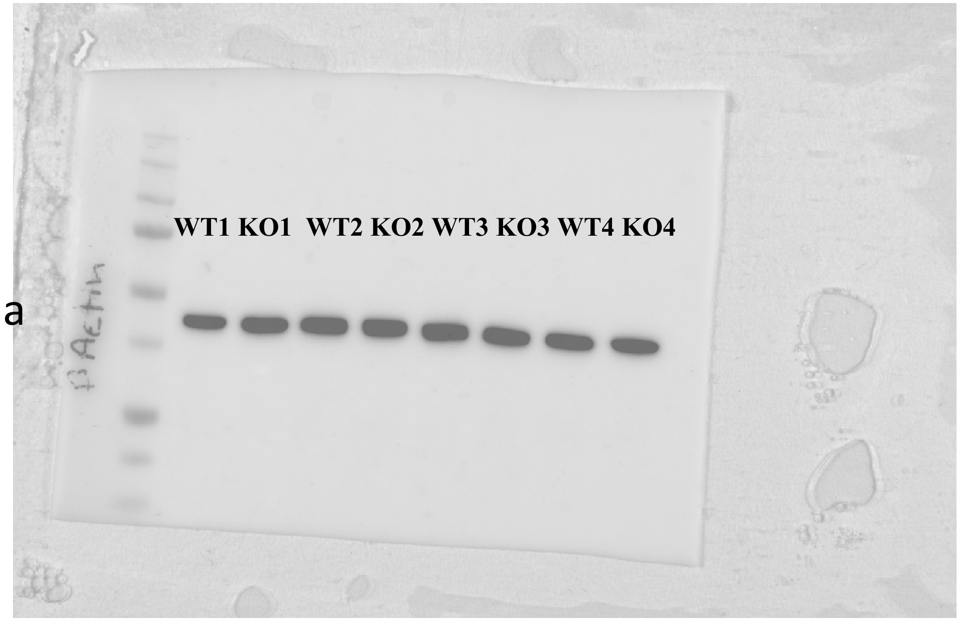

Shank3 Female NR1

105 -120 kda

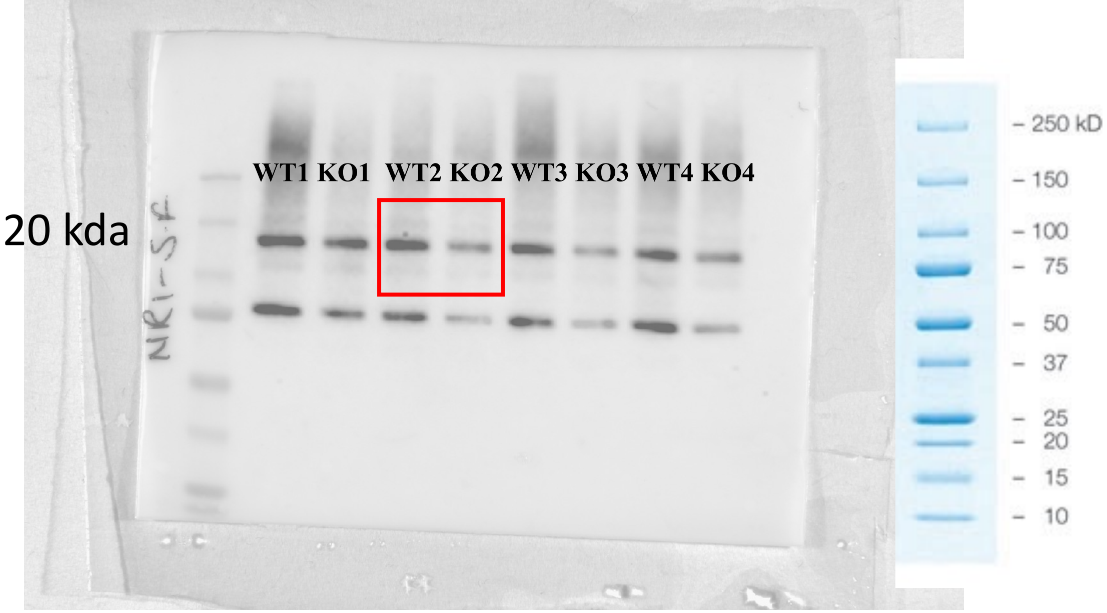

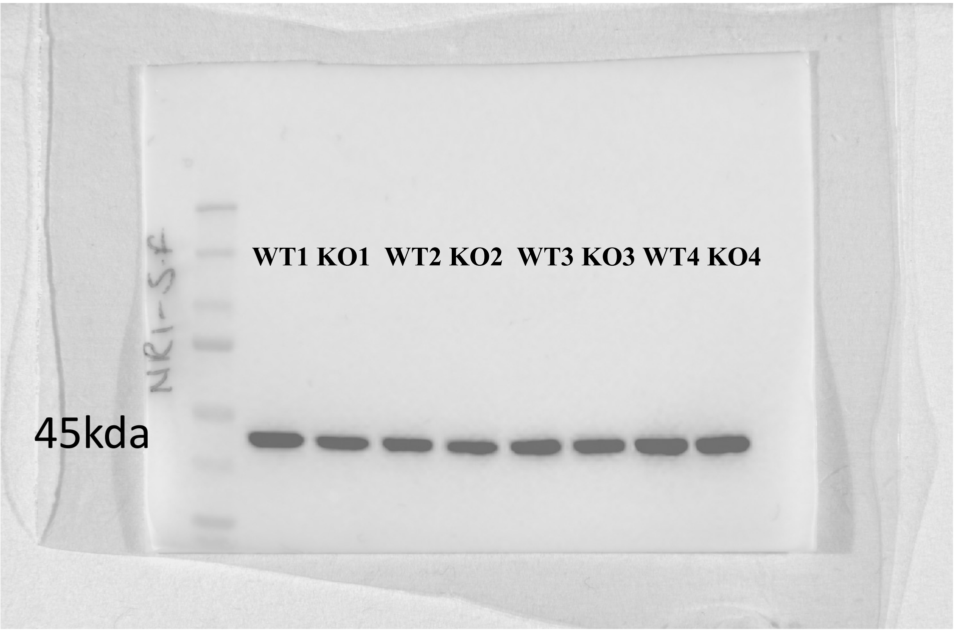

Shank3 Female GAD1

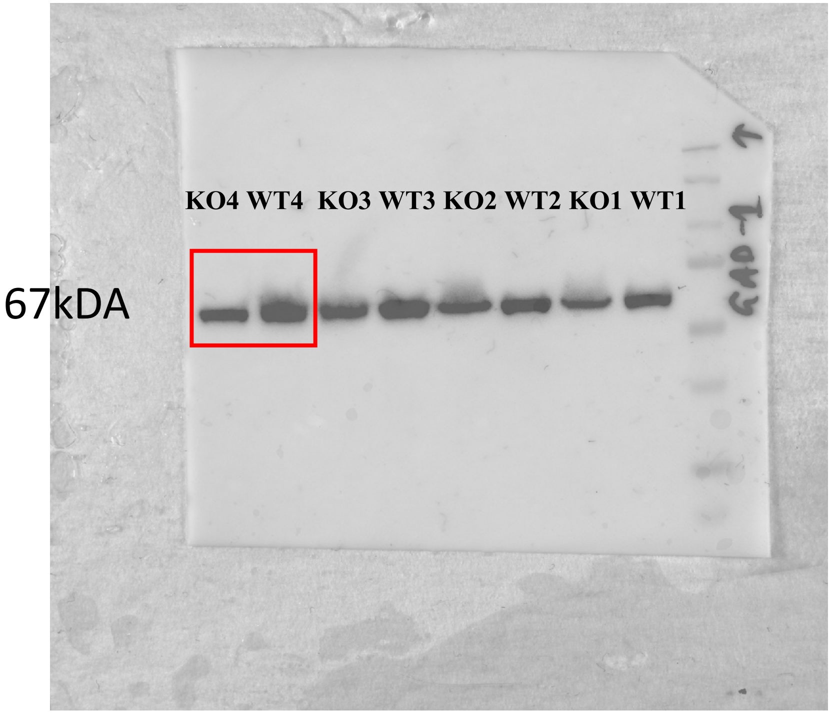

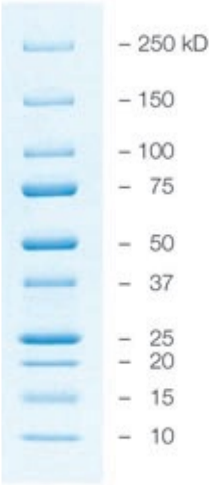

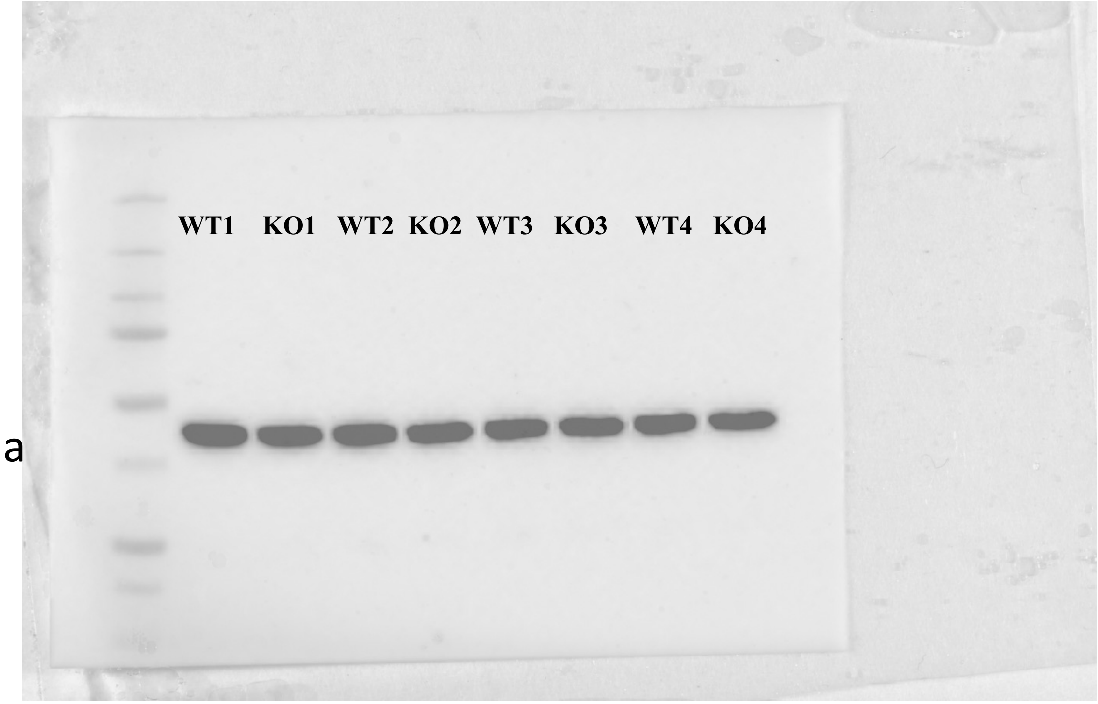

Shank3 Female VGAT

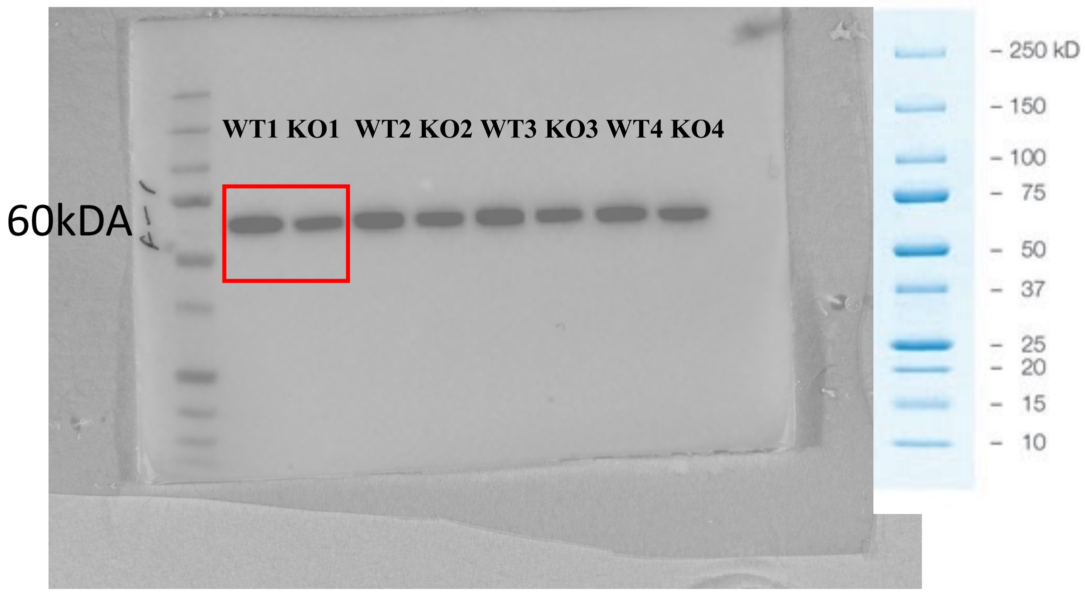

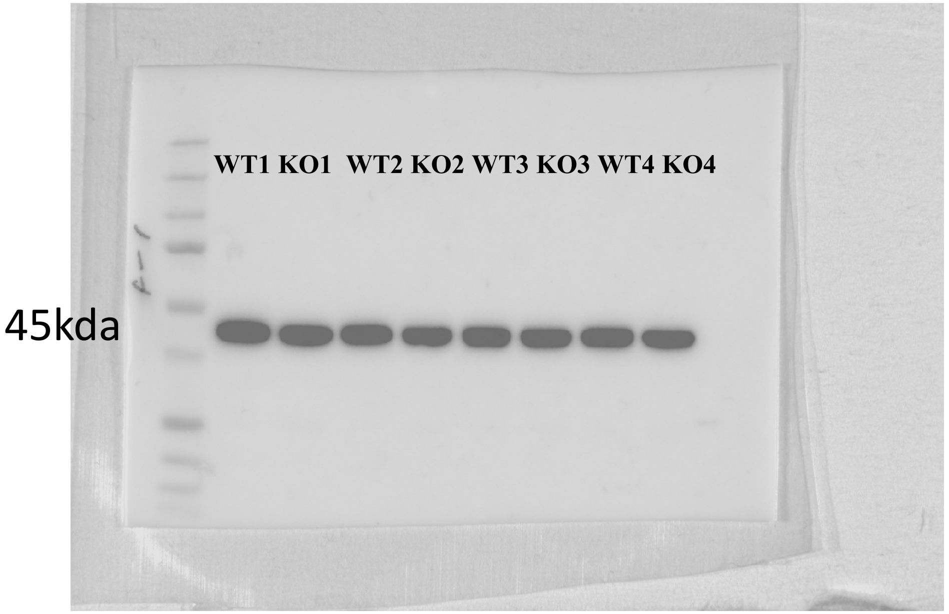

Cntnap2 female group

Cntnap2 female SYP

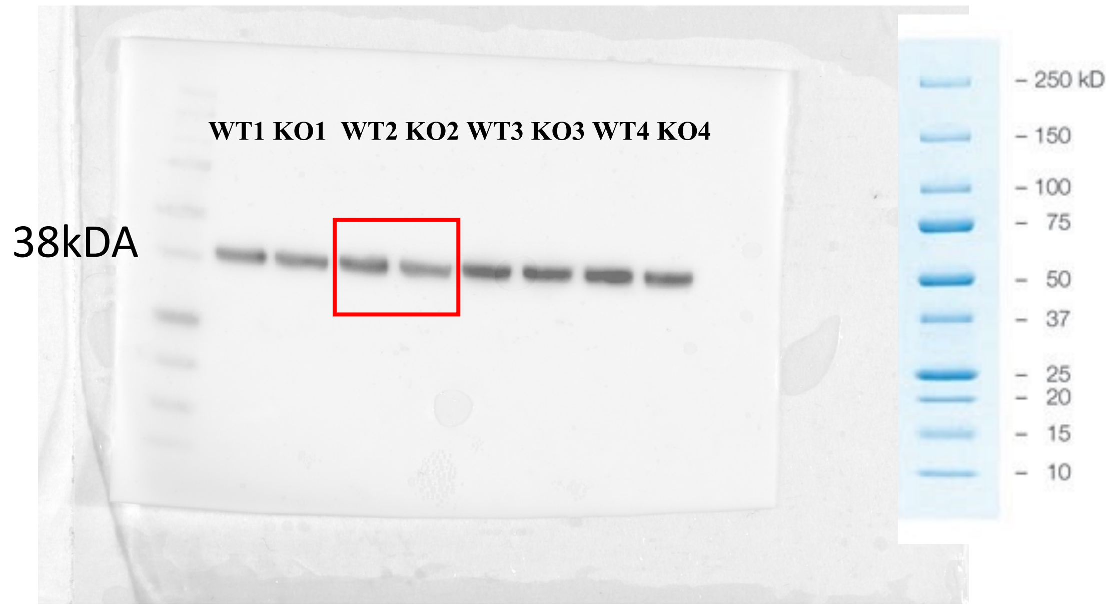

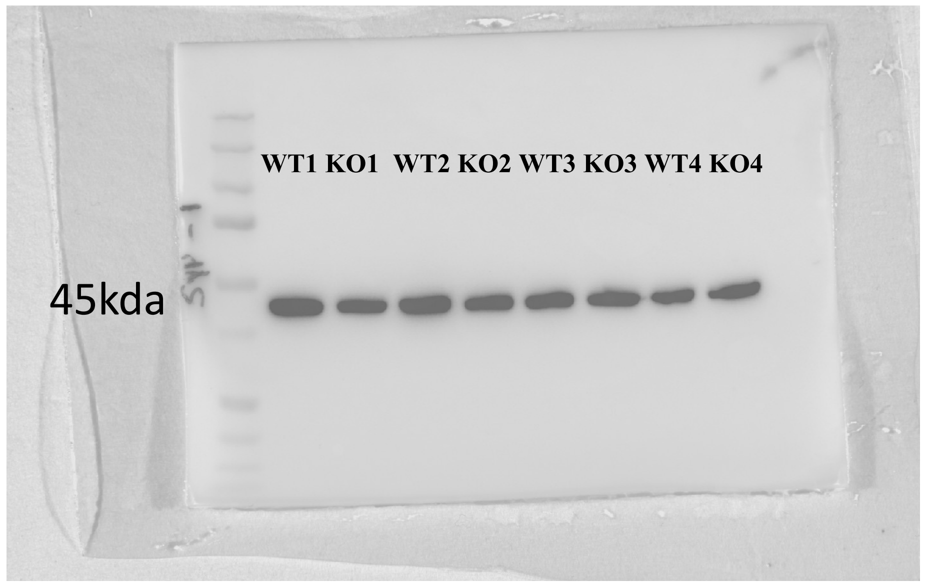

Cntnap2 female NR1

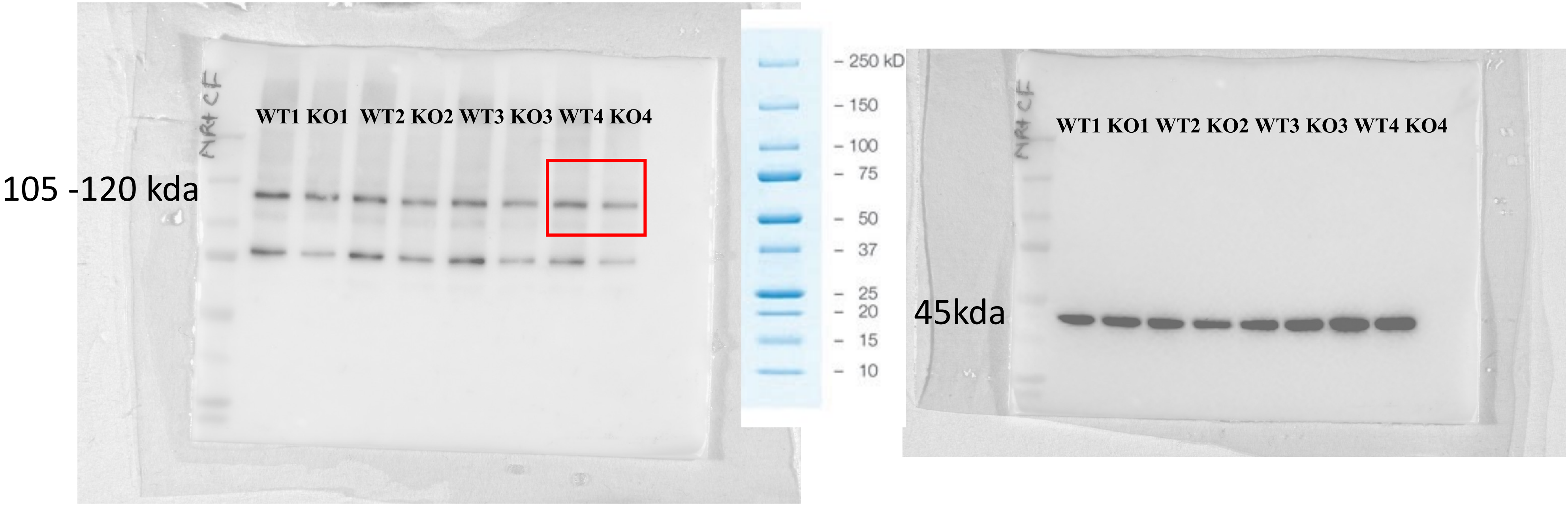

Cntnap2 female GAD1

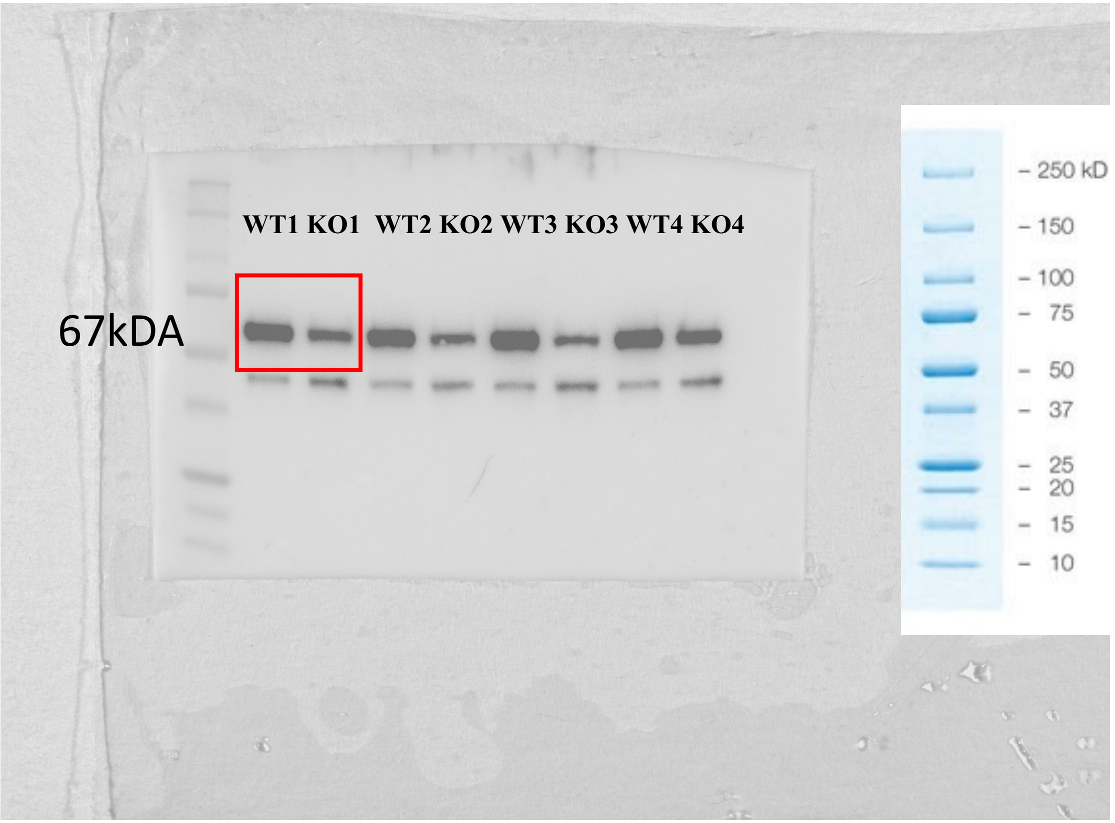

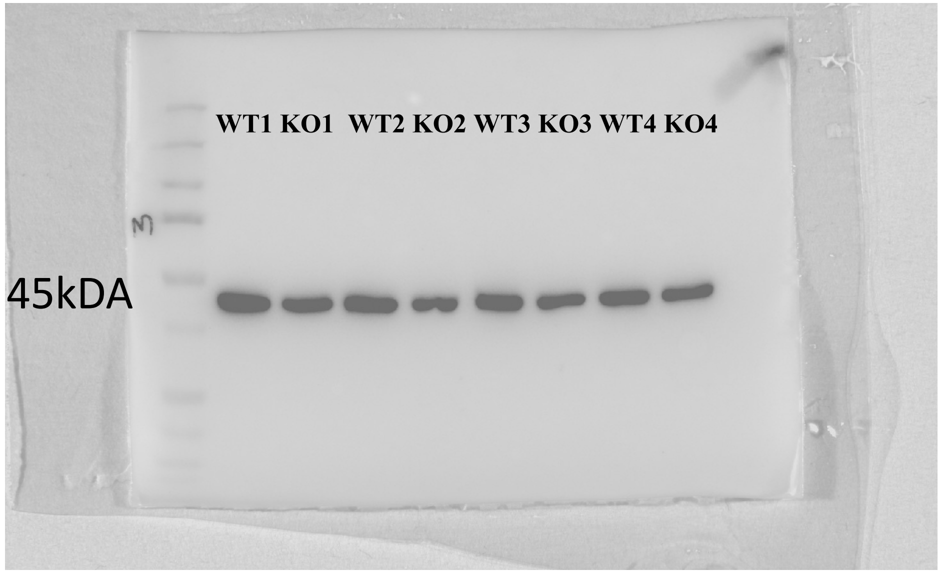

Cntnap2 female VGAT

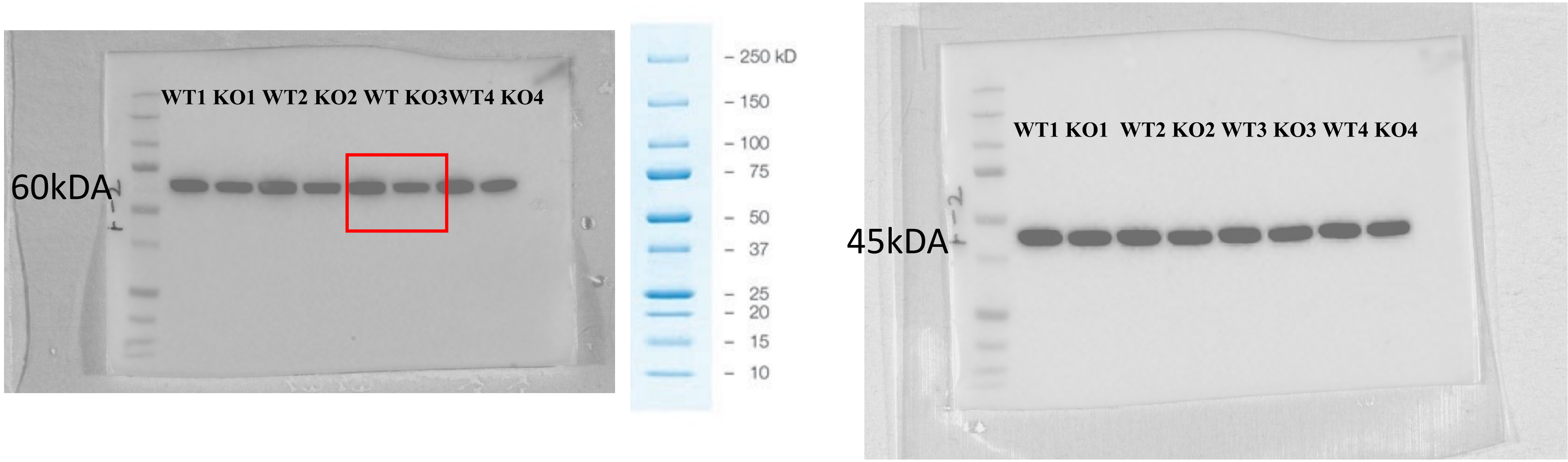

Supplement: Supplementary file 1 — Supplementary Information. [file 41598_2023_50248_MOESM1_ESM.pdf]
